# On the Spleen

**Published:** 1849-01

**Authors:** 


*On the Spleen.* By Drs. Verga and Tigri.—Dr. Verga detailed to the *Scienziati* at Venice the results of his multiplied experiments upon the removal of the spleen of cats, dogs, &c. He arrived at the following conclusions:—1. Nature does not constantly provide animals who have been deprived of their spleens with a new one, nor with a greater development of the liver, the thyroid body, the omentum, or the mesenteric glands. 2. Obesity, salacity, or sterility, are not constant or frequent effects of its removal. 3. Among the least unfrequent phenomena are to be noted during life, a greater vivacity, conjoined with a tendency to tabes, notwithstanding a keen appetite; and after death various alterations in the liver.

Dr. Verga was opposed to considering the spleen as performing a mere mechanical office, as a diverticulum of the blood in the case of impeded circulation; but still he was desirous of giving some account to the Congress of Dr. Tigri's researches, who had discovered in a special condition of the vessels of the human spleen—a mechanism which he termed a "compensator for the circulation." He found as far as the eye and the scalpel could pursue them, that the splenic arteries and veins always ran within a common, inextensible cellular sheath, the veins being four or five times larger than the arteries, and in good part surrounding the calibre of these; the parietes of both vessels being so thin as to allow of the action of the fluid they contained being reciprocally felt. When a too large influx of blood upon the spleen takes place, therefore, the veins compress the arteries and impede a farther flow. Dr. Tigri was surprised to find that in the *horse* the veins and arteries ran at some distance from each other; but this fact, which seemed at first to oppose his theory, was found to support it, when he discovered that Nature, besides having given the vena portæ in this animal a valve, as first shown by Ernest Weber, has likewise furnished the veins leaving the spleen with valves, so that a regurgitation of blood into the viscus is prevented.

Dr. Verga mentioned, that in removing the spleen in cats and dogs, we may divide the duplicature of the peritoneum, connecting it to the stomach, without tying any of the small vessels into which the arteries and veins are there subdivided, these not giving rise to any important hemorrhage.—*Ibid, from Gaz. Medica di Milano*, 1847.

